# Stool filling of an intestinal duplication cyst at the ileocecal valve triggers colonic intussusception: a case report

**DOI:** 10.1186/s40792-018-0527-z

**Published:** 2018-09-15

**Authors:** Sota Kimura, Hiroyuki Iida, Naoto Gunji, Takeshi Gohongi, Takesaburo Ogata

**Affiliations:** 1 Department of Surgery, Tsukuba Gakuen Hospital, 2573-1 Kamiyokoba, Tsukuba, Ibaraki, 305-0854 Japan; 2 Department of Pathology, Tsukuba Gakuen Hospital, 2573-1 Kamiyokoba, Tsukuba, Ibaraki, 305-0854 Japan

**Keywords:** Intestinal duplication, Duplication cyst, Ileocecal valve, Intussusception

## Abstract

**Background:**

Intestinal duplication, a congenital malformation, is considered a rare condition, particularly in adults. Although it affects young children, a minority of patients remains asymptomatic until adulthood. Here, we describe a case of an intestinal duplication cyst that caused intussusception by a unique mechanism.

**Case presentation:**

A 19-year-old man was admitted to our hospital for intermittent abdominal pain. Computed tomography revealed colonic intussusception induced by a nodular mass in the ileocecal region. Urgent ileocecal resection was performed because of the risk of colonic ischemia. The resected material comprised a stool-filled noncommunicating cyst that protruded into the enteric lumen at the ileocecal valve. Histological analyses revealed that the inner wall of the cyst was lined with colonic mucosa and that the muscle layer of the cyst was shared with that of the original enteric wall; furthermore, the cyst had a vestige of an opening site in the wall. We concluded that the cyst was an intestinal duplication that poured stool into its lumen through the tiny orifice, thereby triggering intussusception.

**Conclusions:**

The present case suggests that stool-pouring can cause intussusception into the space of an intestinal duplication lesion.

## Background

Alimentary tract duplication, a congenital malformation, occurs in approximately 1 out of 10,000 births. Ileac or ileocecal duplication is rather common in this condition. More than 80% of these cases are detectable as an acute abdomen or bowel obstruction before 2 years of age; however, a minority of patients remains asymptomatic until adulthood [1–3]. Intestinal duplication has recently attracted attention as a cause of intussusception in young adults [4, 5]. Our present case further elucidates this condition.

## Case presentation

**Fig. 1:**
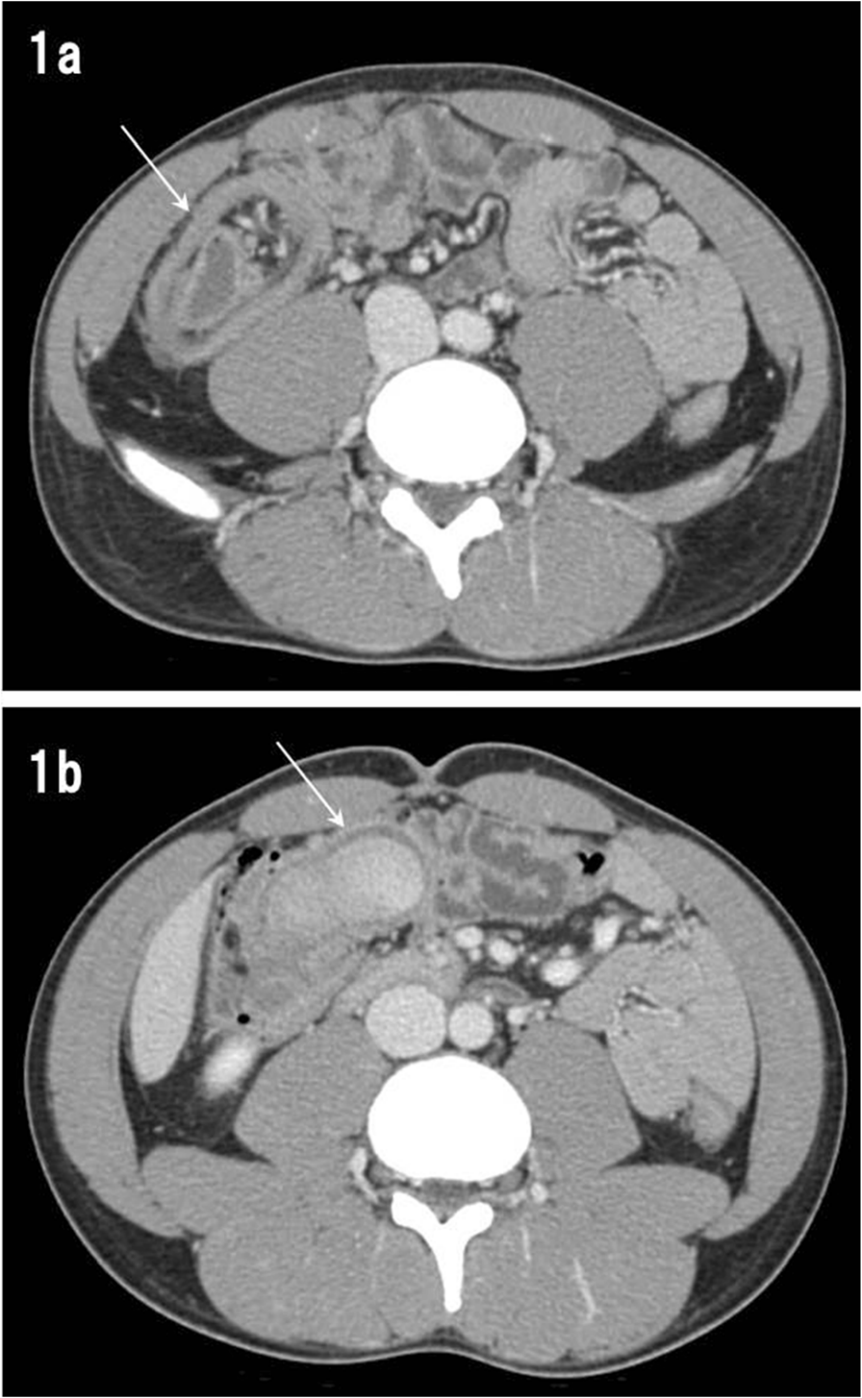
Abdominal computed tomography. **a** A characteristic “target” sign (white arrow) seen in the right abdomen. **b** Tumorous mass (white arrow) acting as a leading point for intussusception

**Fig. 2:**
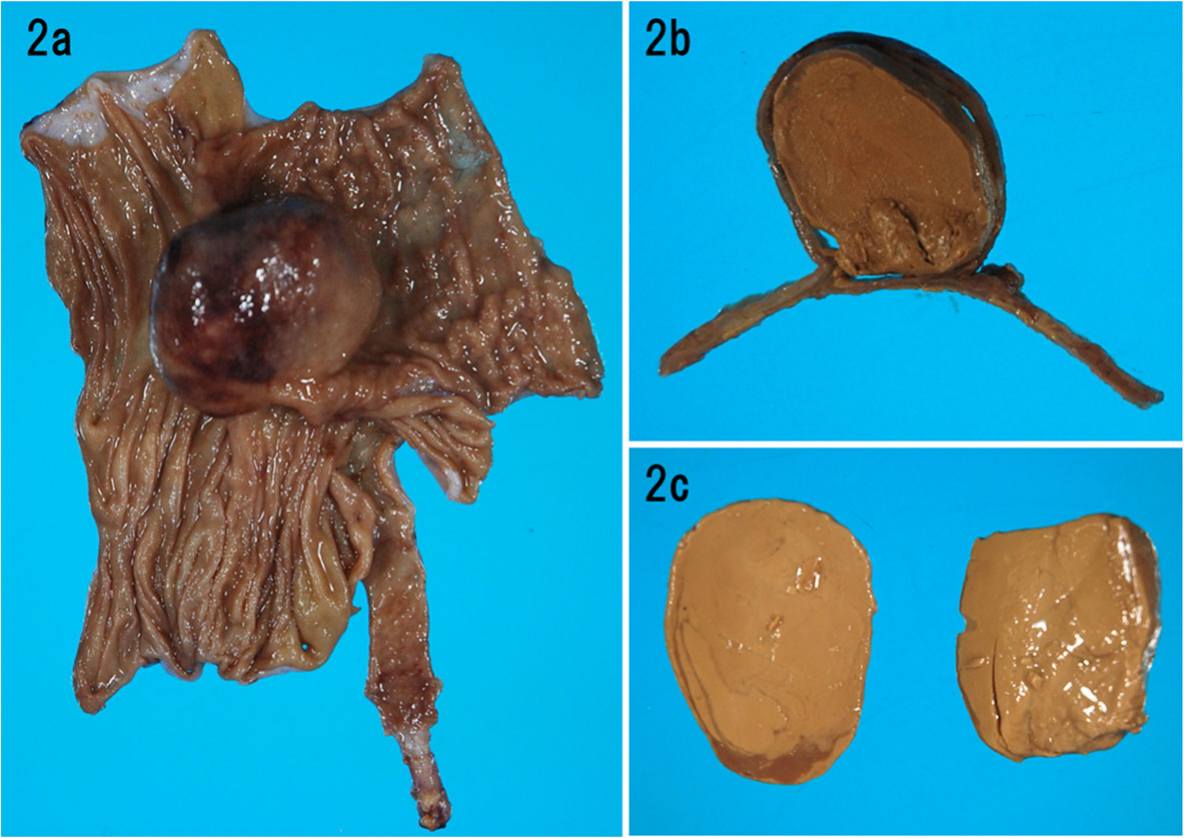
Resected material. **a** An oval duplication cyst of 45 × 35 × 22 mm protruding into the enteric lumen just at the ileocecal valve. **b** The cut surface of the cystic lesion. **c** The cyst was filled with brown-colored stiff stool

**Fig. 3:**
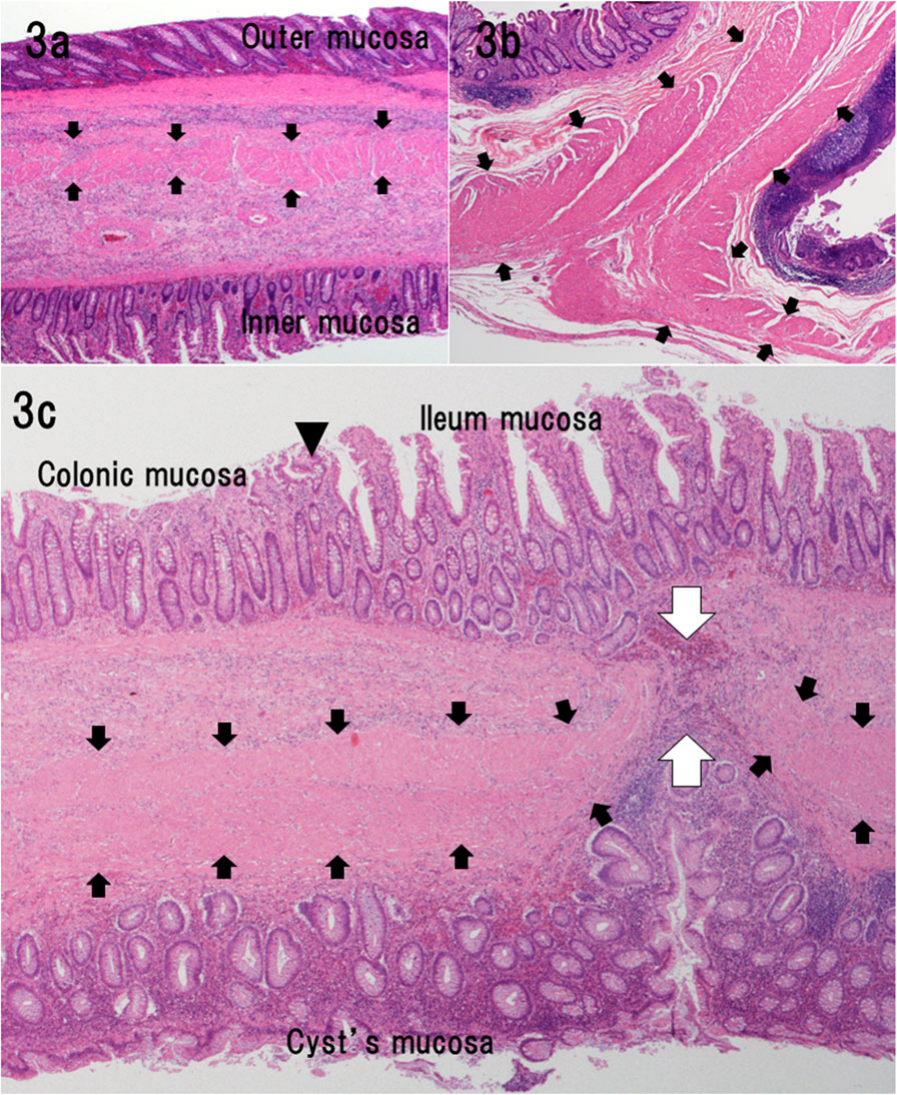
Histopathological findings. **a** The inner wall of the cyst was lined with colonic mucosa, and it shared a muscle layer (arrows) with the original enteric wall. **b** The muscular layer (arrows) was separated into two layers at the edges of the cyst. **c** A vestige of the opening site in the cyst wall: a discontinuity (white arrows) of the cyst’s muscle layer (black arrows) was observed at the top of the cyst near the transitional point (arrowhead) between the ileum and colonic mucosa

A 19-year-old male was referred to our hospital by a primary care physician for a history of intermittent cramping pain in the right flank persisting for several days. His abdomen was flat and soft; however, he complained of abdominal pain upon pressure in the right lower quadrant. Most laboratory test results revealed normal limits except for elevated white blood count (11,170/μL) and serum C-reactive protein level (1.6 mg/dL). Contrast-enhanced computed tomography (Fig. 1) revealed intussusception at the ileocecal region, which appeared as a “target” sign with a tumorous oval mass of 56 × 41 mm as a leading point of intussusception. An urgent operation was performed because of the risk of colonic ischemia due to intussusception. At laparotomy, the bowels had already spontaneously reduced, and a mass was palpable in the ileocecal region. We performed an ileocecal resection, aiming to avoid the potential of relapse of intussusception due to the residual mass. The postoperative course was uneventful and the patient was discharged on day 16 postoperatively. The resected specimen (Fig. 2) comprised an oval cystic lesion of 45 × 35 × 22 mm that protruded into the enteric lumen at the ileocecal valve. The cyst was filled with brown-colored stiff material and did not communicate with the original enteric lumen upon macroscopy. Histological analysis (Fig. 3) revealed that the inner surface of the cyst was completely lined with colonic mucosa and was situated within the intestinal wall of the ileocecal valve. The original muscle layer of the intestine was separated into two layers at the cyst portion and was shared with the muscle layer of the cyst. Thus, we concluded that the cyst was a type of intestinal duplication. Although histological analysis could not identify any opening in the cyst wall, a discontinuity of the muscle layer was observed at the top of the cyst near the transitional point of ileocecal mucosa and the mucosa lined through the hole formed by the lack of muscle layer (Fig. 3c).

### Discussion

Intestinal duplication was first described by Fitz in 1884 [6]; in 1937, Ladd provided a detailed description of the clinical and pathological aspects of duplication of the alimentary tract as a congenital malformation [7]. He defined the three characteristic features of duplication: (1) well-formed smooth muscle layers, (2) an epithelial lining comprising some portion of the alimentary tract, and (3) contiguity with a portion of the alimentary tract. Furthermore, the duplication lesion shares a portion of its wall with that of the adjacent alimentary tract, usually sharing a common blood supply [8]. Duplication of the alimentary tract has been divided into the tubular (14%) and cystic (86%) types. The tubular type often has one or more direct communications with the adjacent bowel, whereas the cystic type usually does not communicate with the lumen of the adjacent bowel and contains a sticky mucoid fluid that is either chocolate or *cafe au lait* colored or almost colorless [7, 9, 10]. Lately, some communicating duplication cysts have been reported in adults [4, 8, 11, 12].

In 2016, Kyo et al. reported a similar cystic lesion filled with stool in an adult colon; however, that cyst was clearly communicating with the original bowel through a tubular twig [4]. They suggested that the cystic feature of duplication was a result of the accumulation of stool in the tubular duplication over the years. In the present case, the duplication cyst was not communicating with the enteric lumen. The cyst content was oval, with a consistency similar to that of stool, and was brown, suggesting that the presence of the stercobilin, which is a metabolic byproduct of bile produced via reduction of bilirubin by bacterial flora in the intestine, is responsible for the brown color of human fecal matter [13]. These findings indicate that the cyst communicated with the alimentary tract before being closed via an unknown mechanism. Our histological study revealed a focal discontinuity of the muscle layer in the cystic wall and a lining of mucosa through a defect in the muscle. Because these features of mucosa, also known as mucosal bridges, were sometimes seen after healing of an ulcer [14, 15] and because there was evidence of previous communication with the original bowel, our histological findings indicated a vestige of the opening site of cystic duplication. Similar to the case reported by Tamvakopoulos et al. [16], it is likely that our case had a directly communicated hole between the cyst and bowel lumen. The hole closed during the healing process, resulting in a discontinuity in the muscular layer and the formation of a mucosal bridge after the stool poured into the cyst through an orifice and subsequently triggered intussusception in adulthood. In adults, intussusception is considered a rare condition and is observed in less than 5% of all cases [17]. More than 80% of these cases are caused by organic lesions, such as inflammatory bowel disease, postoperative adhesions, and benign and malignant tumors. Additionally, malignancy accounts for a maximum of 30% and 66% of intussusception cases occurring in the small and large bowel, respectively [18]. The combination of both intussusception and duplication is rare, especially in adults [4, 5, 11, 12, 19]. These cases have mostly non-specific clinical profile and are difficult to diagnose preoperatively. Because of the large proportion of organic lesions and the significant risk of malignancy, surgical intervention is often required in adult intussusceptions [20]. Regarding the surgical procedure, the possibility of concomitant malignancy should be considered carefully in any case, although prompt intervention is frequently required depending on the clinical symptoms. We decided to perform an emergency surgery considering the possibility of the relapse of intussusception due to mass and the bowel ischemia, which may have led to bowel perforation and peritonitis. Only a few cases have reported on malignant tumors derived from bowel duplication cysts [21–24]; moreover, cases in young adults are extremely rare [25]. The patient’s age and radiological examination findings were not indicative of coexisting malignancy. On conducting laparotomy, regional lymphadenopathy in the mesenterium, adhesion to the peritoneum, or peritoneal seeding was not observed, and ileocecal resection was performed. The patient’s clinical course was uneventful; therefore, the patient was discharged and has been free from abdominal symptoms for more than 5 years since surgery.

## Conclusions

In conclusion, this case of an intestinal duplication cyst in an adult suggests that intussusception could be caused by stool pouring into the space of an intestinal duplication lesion that was not an obstacle in childhood. Although such conditions are extremely rare in adults, they have to be considered for a differential diagnosis of acute abdomen.
